# Customizable Lyophilized Agent for Radiotherapy Imaging and TherapY (CLARITY)

**DOI:** 10.3390/jfb15100285

**Published:** 2024-09-27

**Authors:** Michele Moreau, Debarghya China, Gnagna Sy, Kai Ding, Wilfred Ngwa

**Affiliations:** 1 Department of Radiation Oncology and Molecular Radiation Sciences, Johns Hopkins University School of Medicine, Baltimore, MD 21287, USA; dchina1@jhmi.edu (D.C.); gsy1@jhu.edu (G.S.); kai@jhu.edu (K.D.); 2 Department of Biomedical Engineering, Johns Hopkins University School of Medicine, Baltimore, MD 21218, USA

**Keywords:** lyophilized fiducial markers, CLARITY biomaterial, image-guided radiotherapy (IGRT), immunoadjuvants

## Abstract

Smart radiotherapy biomaterials (SRBs) include seed and liquid biomaterials designed to be employed as fiducial markers during radiotherapy while also delivering therapeutic drug payloads to enhance treatment outcomes. In this study, we investigate a novel Customizable Lyophilized Agent for Radiotherapy Imaging and TherapY (CLARITY) biomaterial, which can be loaded with immunoadjuvants (anti-CD40 monoclonal antibody or Caflanone (FBL-03G)) at the point of care. The CLARITY biomaterial was investigated in an animal model of pancreatic cancer using C57BL6 mice. Mice were imaged before and at different points of time post-treatment to evaluate the potential of CLARITY biomaterial to provide imaging contrast similar to fiducials. This study also used cadavers to assess CLARITY’s potential to provide imaging contrast in humans. Results showed imaging contrast from computed tomography (CT) and magnetic resonance imaging (MRI) modalities for up to 30 days post-treatment, demonstrating potential for use as fiducials. A significant increase in survival (***, *p* = 0.0006) was observed for mice treated with CLARITY biomaterial loaded with immunoadjuvant for up to 10 weeks post-treatment compared to those without treatment. These initial results demonstrate the potential of CLARITY biomaterial to serve as a smart multifunctional radiotherapy biomaterial and provide the impetus for further development and optimization as a point-of-care technology for combination radiotherapy and immunotherapy.

## 1. Introduction

Image-guided radiation therapy (IGRT) uses medical imaging to optimize the precision and accuracy of external beam radiotherapy delivered to hard-to-reach tumors or those located at or near organs at risk [1,2,3]. IGRT routinely utilizes fiducial markers for a more precise alignment of the tumor target area for accurate beam delivery [4,5]. Fiducial markers are routinely used at the clinic to help ensure that radiation therapy is delivered to the targeted tumor region and spares nearby healthy tissue [6,7]. Fiducial markers in the form of seeds, such as gold markers, titanium markers, or liquid such as BioXmark (Nanovi A/S, Denmark) liquid fiducial marker, can provide high precision in targeting the tumor during radiotherapy treatment [8,9,10]. Nonetheless, gold fiducial seeds are known to migrate to different locations and also produce image artifacts. Conversely, liquid fiducial markers such as BioXmark provide exceptional positional stability and visibility [11,12,13,14]. However, current fiducial markers have no additional function besides procuring contrast for image guidance during radiation therapy. Smart radiotherapy biomaterials (SRBs) are currently being developed as multifunctional fiducials designed to provide imaging contrast but also deliver drugs to enhance therapy outcomes [15].

Recent work has focused on development of SRBs designed as seeds or liquid fiducials. Liquid fiducials are designed to address issues posed by gold seed fiducial markers such as migration, image artifacts, and other shortcomings. Although liquid fiducials such as BioXmark can provide excellent image tracking on CT- or MR-based imaging modalities and produce no imaging artifacts, they are not designed to be multifunctional systems that can also deliver drug payloads [9,16]. Recent studies highlighting the application of liquid SRBs loaded with immunotherapeutic drugs in order to provide image guidance during radiotherapy and treat different subcutaneous tumors (e.g., prostate, pancreas, breast, lung, or cervical tumors) have been promising [17,18]. The benefit of SRBs compared to the currently used fiducial markers is that they can provide therapy with minimum toxicity in addition to ensuring geometric accuracy during radiotherapy [18]. SRBs are crafted with high-atomic-number nanoparticles such as gold- or gadolinium-based nanoparticles to fortify impairment to cancer cells via the photoelectric effect [19]. 

Nanogels, which have the consolidated effects of both nanoparticles and hydrogels, such as superior water-binding capacity and prompt reactions to extrinsic instigators such as pH, ions, and temperature, have been used in molecular imaging and as drug delivery systems [20,21]. Previous studies have reported that synthetic or natural polymer-based nanogels encapsulated with gadolinium-based nanoparticles provided enhanced T1-weighted MRI contrast [22,23,24]. Therefore, various nanoparticles have been physically or chemically cross-linked with colloidal polymer hydrogels within the last few decades in nanotechnology in the fight against cancer. Namely, manganese oxide nanoparticles are of interest as nanotheranostics in cancer research due to the fact that manganese oxide nanostructures can deteriorate hydrogen peroxide in the tumor microenvironment into water and oxygen to enhance cancer therapies such as radiotherapy [25]. Manganese oxide nanoparticles can be used as a substitute for gadolinium-based nanoparticles to provide MRI contrast, because manganese oxide nanoparticles can degrade in the tumor microenvironment, delivering Mn^2+^ ions that magnify T1-weighted MRI contrast [25]. This is due to the five unpaired electrons in the 3d orbital of the Mn^2+^ ion, because they construct a substantial magnetic moment that generates adjacent water proton relaxation [26,27].

In this study, we investigate a new design for SRBs, a lyophilized agent that can be readily loaded with a drug payload at the point of care (POC). The Customizable Lyophilized Agent for Radiotherapy Imaging and TherapY (CLARITY) biomaterial is formulated with manganese oxide nanoparticles to provide T1-weighted MRI contrast in addition to CT contrast during image-guided radiation therapy. Advantages of POC technology such as the Customizable Lyophilized Agent for Radiotherapy Imaging and TherapY (CLARITY) biomaterial include the potential to employ such SRBs in low-resource settings, overcoming barriers associated with cold chain, transportation, and stability. In this study, we evaluate the ability of CLARITY biomaterial to serve as a fiducial marker, providing contrast to both computed tomography (CT) and magnetic resonance imaging (MRI) to guide radiotherapy treatment and sustainably deliver therapy-enhancing immunoadjuvants. The investigation was carried out in animal models of pancreatic cancer and cadavers. The significance of developing this technology is to enhance IGRT for cancers such as pancreatic cancer while sparing neighboring normal organs like the stomach and bowel, whose toxicity limits currently prevent the use of ablative biologically effective doses. This technology could be extended for use with other cancers. 

## 2. Materials and Methods

### 2.1. Materials

Different types of nanoparticles were used to produce CLARITY biomaterial to assess which one provides the optimal imaging contrast over time. Manganese Oxide (Mn_2_O_3_) Nanopowder/Nanoparticles Water Dispersion (20 wt%, APS: 30 nm, Purity: 99.2%, Stock #: US3340, Mn_2_O_3_ Nanoparticles SSA: 150 m^2^/g); Cerium Oxide Nanoparticles/CeO_2_ Nanopowder (CeO_2_, Purity: 99.99%, APS:10 nm, Stock #: US3037, Nanoparticles (CeO2) SSA: 35–70 m^2^/g); and Gold Au Nanopowder/Nanoparticles Water Dispersion (GNPs Nanoparticles Aqueous Dispersion, Number of nanoparticles in 1 mL = 3.605 × 10^13^, APS: 14 nm, 1000 ppm, Purple Color, Stock #: US7805, Density of Au is 19.32 g/cm^3^) were obtained from U.S. Nanomaterials Inc. (Houston, TX, USA). All cell culture products (DMEM, RPMI, Trypsin, Fetal Bovine Serum, MEM non-essential amino acids, sodium pyruvate, β-mercaptoethanol, penicillin/streptomycin, and PBS pH 7.4) were acquired from ThermoFisher and Life Technologies (Waltham, MA, USA). A FreeZone 4.5 PLU Liter Benchtop Freeze Dry System from Labconco Corporation (Kansas City, MO, USA) was used to lyophilize the colloid samples to attain the CLARITY biomaterial powder. The monoclonal antibody anti-mouse CD40 (FGK4.5/FGK45) was purchased from BioXcell (New Hampshire, USA). Flavocure Biotech Inc. (Baltimore, MD, USA) supplied the test molecule, Caflanone (FBL-03G), with a purity of 98.7% determined by High-Performance Liquid Chromatography (HPLC).

### 2.2. CLARITY Biomaterial Production

**Figure 1:**
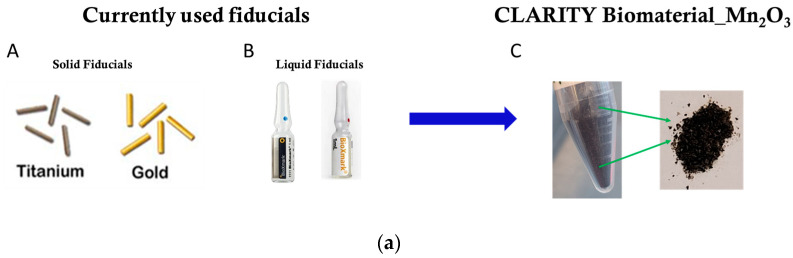

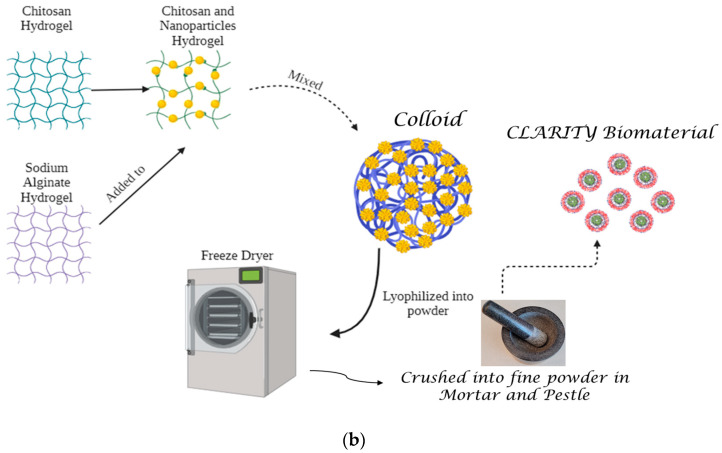
Development of the CLARITY biomaterial. (**a**) The evolution of smart radiotherapy biomaterials is shown in Figure A (Radiation Products Design, Inc. Albertville, MN, USA) and Figure B (BioXmark, Nanovi A/S, Denmark), which depicts currently-used solid and liquid fiducials, respectively, which are being replaced by multifunctional smart biomaterials. Figure C depicts CLARITY biomaterial, formulated with nanoparticles such as manganese oxide nanoparticles, as they can provide imaging contrast over the duration of radiotherapy treatment and can also deliver drugs in situ at the tumor site. (**b**) Illustration of CLARITY biomaterial production is shown in detail [18]. Two natural polymers are mixed in a 1:1 ratio, incorporating imaging-contrast nanoparticles such as manganese oxide (Mn_2_O_3_) nanoparticles [18]. The frozen colloid is lyophilized. The lyophilized product is then crushed into a fine powder using a mortar and pestle. The resulting powder has different size distributions of the CLARITY biomaterial that can be reconstituted with the immunoadjuvant drug at a point of care and administered to subjects.

A total of 2% (*w*/*v*) Chitosan hydrogel was prepared in 1% acetic acid in a vial, and 4% (*w*/*v*) sodium alginate hydrogel was prepared in deionized water in a separate vial. The 2 hydrogels were mixed in a 1:1 ratio and mixed with 1:½ of solution made using either manganese oxide, cerium oxide, or gold nanoparticles to provide imaging contrast for radiotherapy treatment. This mixture of hydrogels and nanoparticles was aliquoted into falcon tubes and allowed to initially freeze at −80 °C for at least 24 h before lyophilization. The frozen samples were then lyophilized using a freeze dryer at −88 °C and 0.12 mBar for up to 72 h. The lyophilized sample from the freeze dryer mimics the shape of the falcon tube container and resembles a meshy dry substance that crumbles upon touch. The lyophilized samples were then pulverized in a mortar and pestle to obtain a powder, as illustrated in Figure 1a,b below. The lyophilized samples, now the CLARITY biomaterial, were then rehydrated using an immunoadjuvant drug solution. Once the CLARITY biomaterial was rehydrated with the immunoadjuvant solution, it was sonicated for 5 min to obtain a more homogenized solution.

### 2.3. Nanoparticle Tracking Analysis (NTA)

The size distribution of the CLARITY biomaterial was assessed in a solution containing the immunoadjuvant drug. The size distribution provides an initial assessment of the range of sizes found when the powder is rehydrated with either anti-CD40 or Caflanone. Particle quantification was performed using the ZetaView BASIC NTA-Nanoparticle Tracking Video Microscope PMX-120 (Particle Metrix, Mebane, NC USA) in scatter mode with the following capture settings: sensitivity 60–65, shutter 100, and minimum trace length 10. Capture was performed at medium video settings, corresponding to 30 frames per position. ZetaView software version 8.5.10 was used to analyze the recorded videos with the following settings: minimum brightness 30, maximum brightness 255, minimum area 10, and maximum area 1000.

### 2.4. Animal Studies with KPC Syngeneic Pancreatic Cancer Cells

KPC cell lines derived from an LS-Kras; p53+/floxed, Pdx-cre mouse were utilized. The pancreatic cancer cell line, KPC, obtained from the ATCC, was cultured in Dulbecco’s Modified Eagle’s Medium (DMEM) with 5% Fetal clone II FBS and 1% penicillin/streptomycin. All cells were cultured at 37 °C in a humidified incubator with 5% CO_2_. Immunocompetent wild-type C57BL/6-strain female mice were acquired from Charles River at 6 weeks old. They were inoculated subcutaneously 4 weeks following their arrival with 9.4 × 10^4^ pancreatic cancer cells with a volume of 100 μL of cells per flank. Following cancer cell injection, the tumors were allowed to grow over 2 weeks to at least 3.0 mm or greater in diameter size before the start of treatment. Animal experiments followed the guidelines and regulations set by the Johns Hopkins University Animal Care and Use Committee (ACUC) under approved protocol # MO21M281. Mice maintenance in the Johns Hopkins University animal facility was performed under the Institutional Animal Care and Use Committee-approved guidelines.

### 2.5. Pancreatic Tumor Mice CT and MR Imaging

Mice were anesthetized with isoflurane before any computed tomography or magnetic resonance images were taken. In vivo whole-body CT imaging (nanoScan PET/CT, Mediso Medical Imaging Systems, Arlington, VA, USA) was performed in the Center for Infection and Inflammation Imaging Research (Ci3R) at the Johns Hopkins University School of Medicine. Mice bearing pancreatic (KPC) subcutaneous tumors inoculated with 100 μL of the CLARITY biomaterial, formulated with Mn_2_O_3_ and loaded with 800 µg of Caflanone, were imaged using 50 kVp X-ray voltage which was reconstructed to 0.16 mm^3^ sized voxels. CT images were visualized with Vivoquant 2020. All MR images were acquired on a multi-nuclear BioSpec 70/30 PET-MR 7T scanner (Bruker Biospin MRI Inc., Billerica, MA, USA). The mice were anesthetized under ~1.5–2% isoflurane. First, quick whole-body coronal images were acquired to localize the tumor using T2-weighted fast spin-echo rapid imaging with a refocused echo (RARE) sequence (repetition time, 3 s; echo time, 30 ms; RARE factor, 8; number of averages, 1). T1 weighted axial, coronal, and sagittal images were then acquired with a RARE sequence (repetition time, 600 ms; echo time, 7 ms; RARE factor, 2; the number of averages, 6; matrix size, 133 × 133; field of view, 3.2 × 3.2 cm; slice thickness, 1 mm; in-plane resolution, 240 × 240 μm, slice numbers, 20 to 25). 

### 2.6. MRI and CT Acquisition for the Human Cadaver

To further assess the feasibility of the CLARITY biomaterial in providing image guidance, contrast imaging was conducted in a human cadaver approved by the Johns Hopkins Institutional Review Board (IRB) under protocol # NA_00070589 (PI KD). The source of the human cadaver involved in our research is the State of Maryland Anatomy Board. As an academic center, we can send requests to get and then later return the cadaver from the State of Maryland Anatomy Board for medical education and research. Please see this link https://health.maryland.gov/anatomy/Pages/EducatorsResearchers.aspx (accessed on 25 July 2024) for more information. A refrigerated, unfixed cadaveric specimen was implanted in 2 regions: (a) the right kidney, with 3 mL of the CLARITY biomaterial formulated with manganese oxide nanoparticles, and (b) the breast, with 3 mL of the CLARITY biomaterial formulated with cerium oxide nanoparticles. CT simulation (TOSHIBA Helical CT scan with 2 mm slice thickness, 120 kVp, and X-ray tube current of 100 mA) was done with the cadaveric specimen in the supine position. The human cadaver specimen was subsequently imaged again. A Philips Achieva 3.0 T MRI System with BODY Transmit Coil was used for MRI acquisition with a repetition time of 5.31 ms, a flip angle of 100, a percent phase field of view of 70.833, and a slice thickness of 0.9 mm. CT and MR imaging were done before and after injection of the CLARITY biomaterial formulations.

### 2.7. Electron Microscopy Analysis

#### Transmission Electron Microscopy (TEM): Negative Stain

Samples (8 µL) were adsorbed to glow-discharged (EMS GloQube) ultra-thin (UL) carbon-coated 400 mesh copper grids (EMS CF400-Cu-UL) by floatation for 2 min. Grids were rinsed in 3 drops (5 s each) of TBS (Tris-buffered saline) and negatively stained in 2 consecutive drops of 1% uranyl acetate (UA, aq.) and quickly aspirated. Grids were imaged on a Hitachi 7600 TEM operating at 80 kV with an AMT XR80 CCD (8 megapixels).

### 2.8. Nanoparticles Used for Image Guidance

In addition to manganese oxide (Mn_2_O_3_), cerium oxide (CeO_2_) and gold (GNPs) nanoparticles were used to provide imaging contrast in either the mice’s subcutaneous tumors or the human cadaveric specimen.

### 2.9. Statistical Analysis

Group pairs were compared using a two-tailed Student’s *t*-test. All reported tests were two-tailed and were considered significant (ns, means not substantial, * p < 0.05; *** p < 0.001). Survival assays were plotted using GraphPad Prism and were analyzed using Log-rank (Mantel-Cox) and Gehan-Breslow Wilcoxon tests. Error bars are SD unless otherwise noted.

## 3. Results

### 3.1. Characterization of the CLARITY Biomaterial

**Figure 2:**
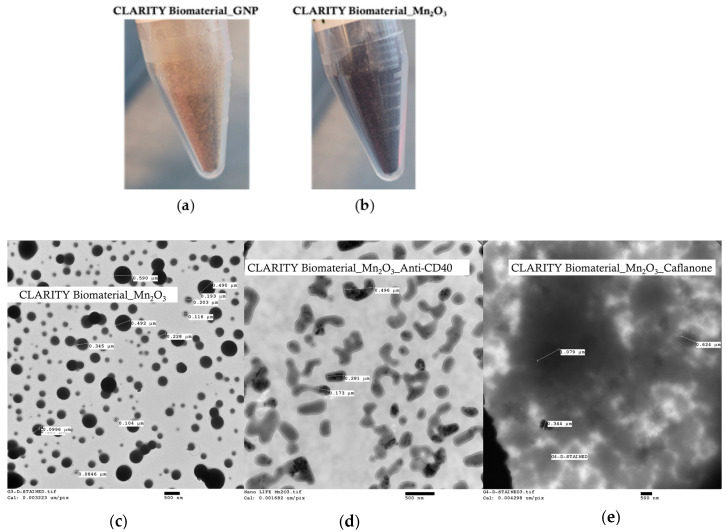

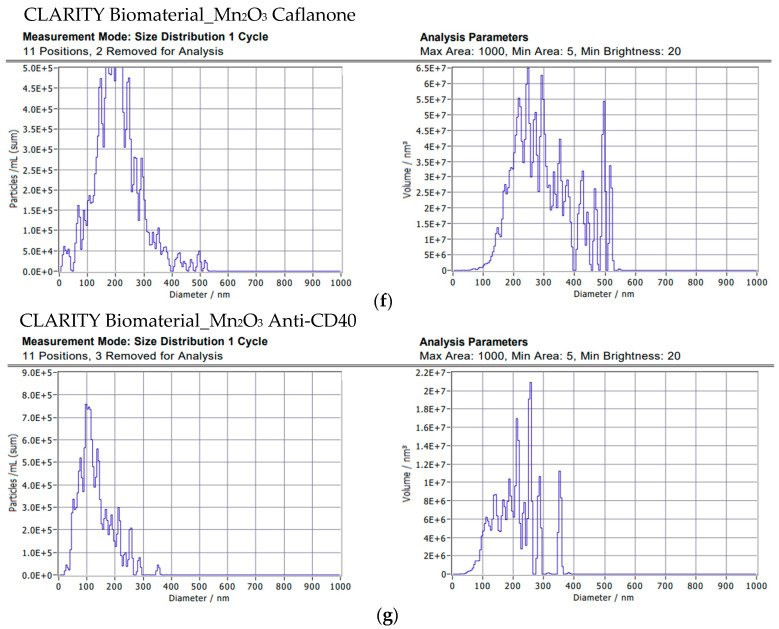
Depiction of CLARITY biomaterial in its natural form. (**a**) CLARITY biomaterial formulated with gold nanoparticles (GNP), (**b**) CLARITY biomaterial formulated with manganese oxide nanoparticles (Mn_2_O_3_). Electron microscopy images of CLARITY biomaterial are also depicted to show its size and morphology. (**c**) Transmission electron microscopy images of CLARITY biomaterial_Mn_2_O_3_ displaying the sizes of its spherical shapes (500 nm). (**d**) TEM images of CLARITY biomaterial_Mn_2_O_3_ loaded with anti-CD40 monoclonal antibody. (**e**) TEM images of CLARITY biomaterial_Mn_2_O_3_ loaded with Caflanone. (**f**,**g**) The nanoparticle tracking analysis (NTA) provided polydisperse nanosized particles for CLARITY biomaterial samples tested. The NTA enabled accurate sizing and a clear distinction of the size populations for CLARITY biomaterial_Mn_2_O_3_ Caflanone (mean size = 207 nm) (**f**) and CLARITY biomaterial_Mn_2_O_3_ Anti-CD40 (mean size = 130 nm) (**g**). The NTA Supplementary Files highlight the range of size populations for the CLARITY biomaterial loaded with either Caflanone or anti-CD40. Note: for Figures (**f**,**g**), the numbers on the y-axis have a notation of ‘E’ which means ‘× 10’ for example ‘8.0E+5’ means ‘8.0 × 10^5^’.

Illustrations of the CLARITY biomaterial in its natural powder form, formulated with either gold nanoparticles (GNP) or manganese oxide nanoparticles (Mn_2_O_3_), are displayed in Figure 2a and Figure 2b, respectively. Transmission electron microscopy images are shown in Figure 2, highlighting the size and micromorphology of the CLARITY biomaterial formulated with manganese oxide (Mn_2_O_3_) nanoparticles (Figure 2c and Supplementary Figure S1) or loaded with either anti-CD40 monoclonal antibody (Figure 2d and Supplementary Figure S1) or Caflanone (Figure 2e and Supplementary Figure S1). Nanoparticle tracking analysis of the CLARITY biomaterial_Mn_2_O_3_ reconstituted with either Caflanone or anti-CD40 showed a clear distinction in its size distribution in solution for the Caflanone (size range = 100–300 nm) (Figure 2f) versus the anti-CD40-loaded CLARITY biomaterial (size range = 63–210 nm) (Figure 2g). A detailed size distribution range is also displayed in the NTA Supplementary Files. 

### 3.2. Image Guidance of CLARITY Biomaterial in Pancreatic Cancer

**Figure 3:**
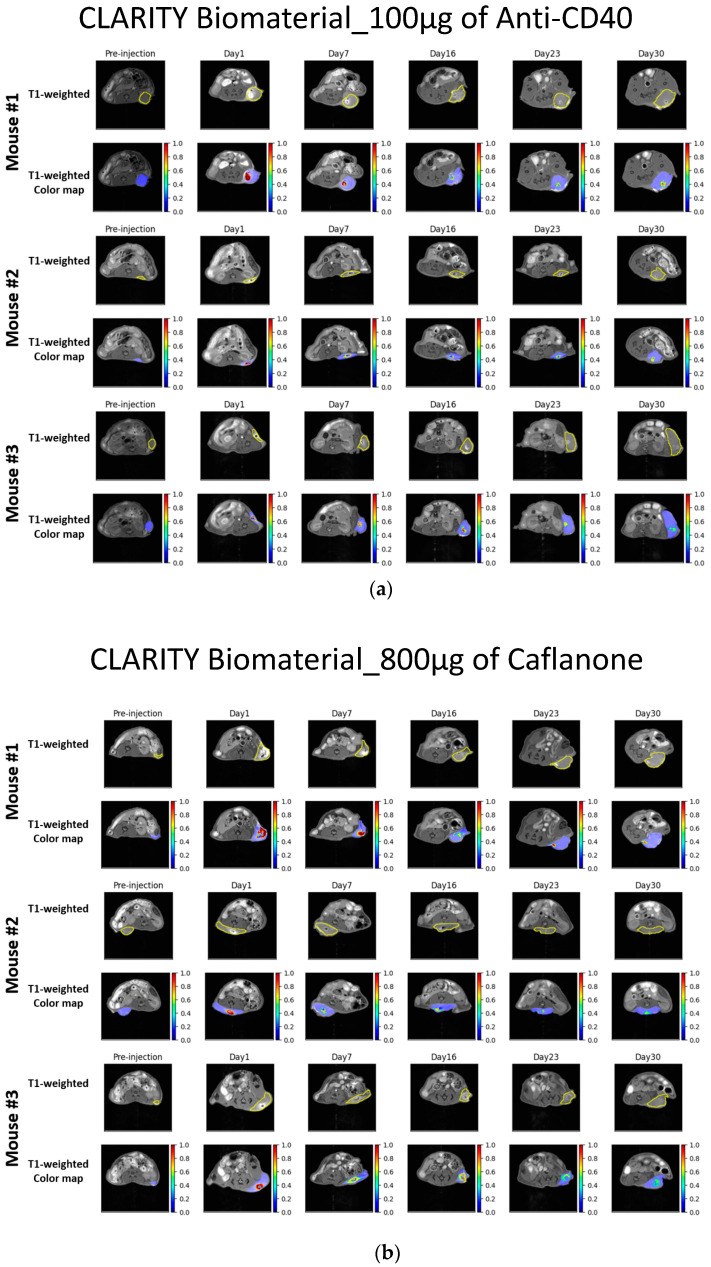

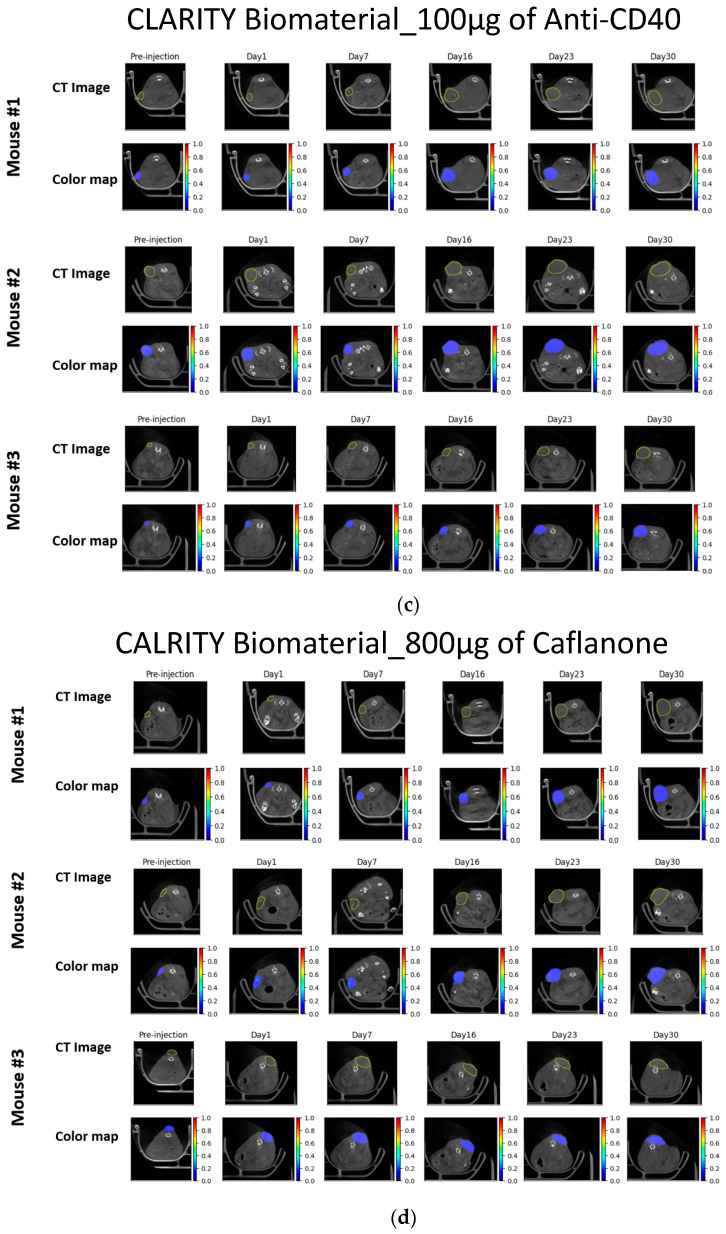
Image guidance of CLARITY biomaterial in MR and CT imaging modalities. (**a**) MR images of mice bearing subcutaneous pancreatic cancer that received 100 µg of anti-CD40-loaded CLARITY biomaterial. (**b**) MR images of mice bearing subcutaneous pancreatic cancer that received 800 µg of Caflanone-loaded CLARITY biomaterial. (**c**) CT images of mice bearing subcutaneous pancreatic cancer that received 100 µg of anti-CD40-loaded CLARITY biomaterial. (**d**) CT images of mice bearing subcutaneous pancreatic cancer that received 800 µg of Caflanone-loaded CLARITY biomaterial.

This study focused on investigating a lyophilized powder that can be customized using different immunoadjuvants to provide image guidance and also boost therapeutic outcomes in pre-clinical mouse models of pancreatic cancer. The CLARITY biomaterial was evaluated for providing both CT and MRI contrast over time when administered in pancreatic tumor tissue. Figure 3a,b highlight the image guidance capability of the CLARITY biomaterial in providing MRI contrast for up to 30 days post-treatment. Figure 3c,d display the CT contrast provided by the CLARITY biomaterial for up to 30 days post-treatment, at which point the contrast was no longer visible.

**Figure 4:**
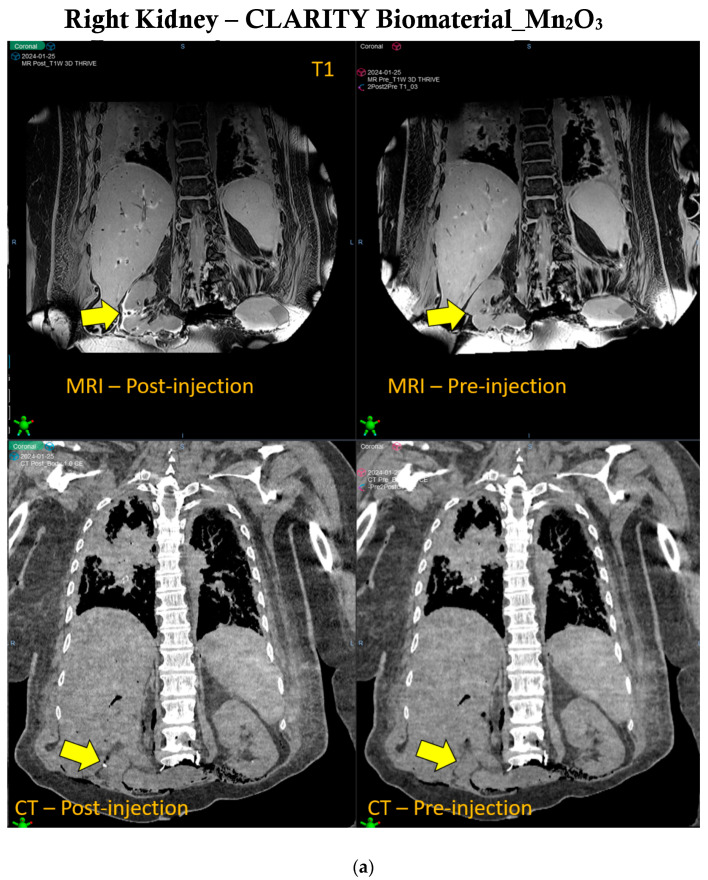

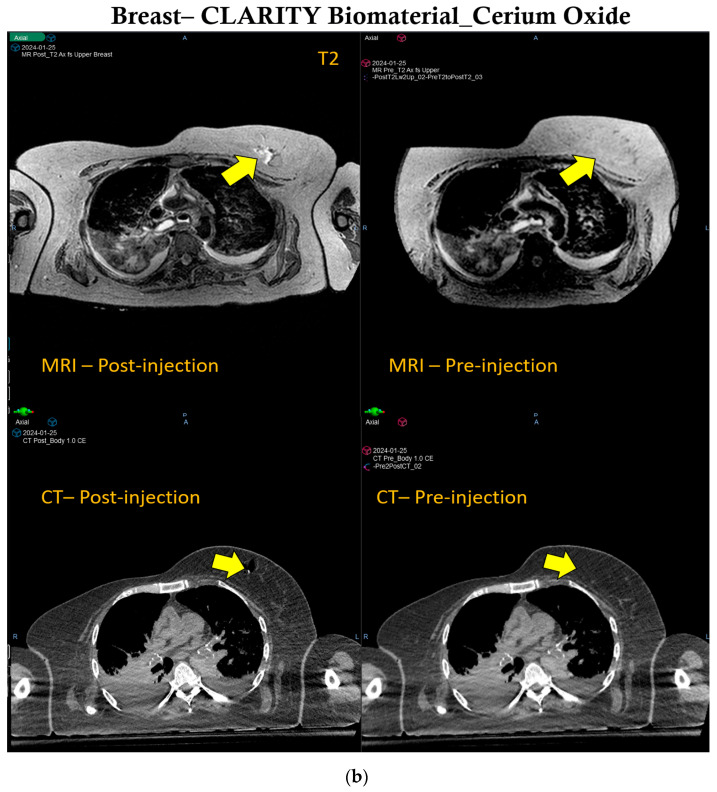
IGRT capability of CLARITY biomaterial in Human Cadaver. (**a**) The right kidney of the human cadaver specimen is showing CT and MRI (T1-weighted) contrast after being inoculated with CLARITY biomaterial formulated with manganese oxide nanoparticles, as shown by the yellow arrow. (**b**) The breast tissue is showing similar CT and MRI (T2-weighted) contrast post-injection of the CLARITY biomaterial formulated with cerium oxide nanoparticles, as indicated by the yellow arrow.

Manganese oxide offers significant multi-functionality in the CLARITY biomaterial, providing both CT and MRI contrast over time during radiotherapy. Figure 3 shows that the contrast provided by manganese oxide nanoparticles is brighter in MR images and lasts beyond the 30 days of observation, as opposed to the CT contrast provided by these same nanoparticles. However, Supplementary Figure S2 shows CT contrast for up to 30 days post-treatment. Human cadaveric specimens were used to further evaluate the CT and MRI contrast provided when the CLARITY biomaterial was formulated with Mn_2_O_3_ and injected into the right kidney of the specimen, as indicated by the yellow arrow shown in Figure 4a. CLARITY biomaterial formulated with cerium oxide interestingly also showed both CT and MRI contrast in the breast tissue of the specimen, as shown in Figure 4b. An additional functionality of the manganese oxide nanoparticles is the potential to address hypoxia, which is critical for enhancing radiotherapy and overcoming immunosuppression [28,29,30,31].

### 3.3. Efficacy of CLARITY Biomaterial in Pancreatic Cancer

**Figure 5:**
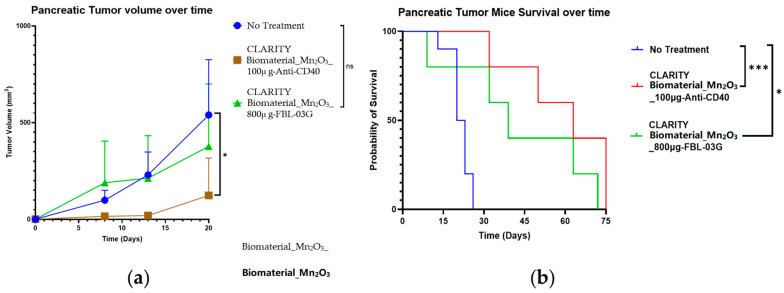
Efficacy of CLARITY biomaterial in pancreatic cancer. C57BL6 mice bearing subcutaneous pancreatic tumors were treated with CLARITY biomaterial_Mn_2_O_3_ loaded with either 100 µg of Anti-CD40 (*n* = 5) or 800 µg of Caflanone (*n* = 5) and were compared to a no-treatment group (*n* = 10). (**a**) Significantly delayed tumor growth was observed for mice treated with CLARITY biomaterial loaded with 100 µg of Anti-CD40 (*, *p* = 0.0212) compared to those treated with CLARITY biomaterial loaded 800 µg of Caflanone (ns, *p* = 0.4165) or the no-treatment group. (**b**) Significantly prolonged survival (***, *p* = 0.0006) was observed for mice treated with CLARITY biomaterial loaded with 100 µg of Anti-CD40 compared to those treated with CLARITY biomaterial loaded with 800 µg of Caflanone (*, *p* = 0.0124) or the no-treatment group.

The CLARITY biomaterial was evaluated for its therapeutic capability when loaded with either 100 µg_anti-CD40 monoclonal antibody or 800 µg_Caflanone drug against a no-treatment group of C57BL6 mice bearing subcutaneous pancreatic tumors. Significant delay in tumor growth was observed for the group that received CLARITY biomaterial_Mn_2_O_3_ loaded with 100 µg of anti-CD40 (*, *p* = 0.0212) compared to the no-treatment group (Figure 5a). In addition, significant rates of survival were also observed for the cohorts treated with either the CLARITY biomaterial loaded with 100 µg of Anti-CD40 (***, *p* = 0.0006) or the CLARITY biomaterial loaded with 800 µg of FBL-03G (*, *p* = 0.0124) compared to the no-treatment group (Figure 5b).

These results highlight the therapeutic potential of the CLARITY biomaterial formulated with manganese oxide nanoparticles and loaded with drugs. The potential of the CLARITY biomaterial to serve as a fiducial, and to induce significant outcomes has been demonstrated in pancreatic cancer with prolonged mice survival observed for the treated cohorts compared to the no-treatment cohort. 

## 4. Discussion

Pancreatic cancer is one of the most lethal cancers, with a 5-year survival rate of 13% according to the American Cancer Society, Cancer Facts & Figures 2024 [32,33]. Despite advances in diagnosis and treatment, the incidence and mortality of pancreatic cancer have increased steadily, and it is now projected to become the second leading cause of cancer death by 2030 [34,35]. This is driven by the fact that pancreatic cancer is often diagnosed at the metastatic stage, and pancreatic tumors are notoriously resistant to therapy [36]. Major disparities also exist with some populations experiencing both higher incidence and death rates from pancreatic cancer than other populations, partly driven by limited access to effective treatment [37,38]. Therefore, there is a critical unmet need for new treatment approaches to improve survival and reduce disparities. The potential significance of this project will be to meet this urgent need by developing a treatment option for significantly increasing survival and reducing disparities. 

The new CLARITY biomaterial relates to fiducial markers, devices used to provide imaging contrast during image-guided radiotherapy. In radiation therapy (RT), fiducials allow for accurate and precise targeting of tumor sites during RT, ensuring that RT is delivered to the tumors while minimizing any radiation to neighboring healthy tissues or organs at risk [1,2,3,4]. Currently, there are two types of fiducial markers used in the clinic. These include solid fiducials and liquid fiducials. Limitations of solid fiducial markers made of materials such as gold, titanium, etc., include the fact that they could migrate, require specific size (gauge) needles for administration, and have the potential for producing imaging artifacts or perturbing the RT dose [39,40]. Solid fiducials are also limited to organs like tissue boundaries, e.g., the duodenum wall and bladder wall, thus, only liquid fiducial is suitable there. Over the last few decades, polymer-based fiducial markers, either solid or liquid, have been developed to address some of these issues. Although polymer-based liquid fiducials can provide excellent image tracking on CT-based imaging modalities in addition to producing no imaging artifacts, they have limitations in storage temperatures, which may not be suited for low-resource settings, and they also do not have the capacity for drug delivery [9,16]. 

This study has demonstrated the potential of CLARITY biomaterial formulated with CT and/or MRI contrast nanoparticles to provide computed tomography and magnetic resonance bimodal imaging contrast over several weeks post-inoculation. No significant image artifacts were observed from the CT or MR images shown in Figure 3 and Figure 4 and Supplementary Figure S2. Another observation as it relates specifically to CT contrast is the fact that the amount of CLARITY biomaterial formulated with manganese oxide nanoparticles used to reconstitute with the drug needs to be at least 20 mg in mass in order to produce CT contrast over time (Supplementary Figure S2). There was no visible CT contrast seen in Supplementary Figure S2a, while the possibility of a threshold for CLARITY biomaterial_Mn_2_O_3_ needed to yield visible CT contrast is observed in Figure S2b. This study did not evaluate any potential toxicity or side effects related to this mass threshold which can be addressed in future studies. Hence, there is flexibility in managing the contrast capability of the CLARITY biomaterial throughout the radiotherapy treatment due to the amount of manganese oxide nanoparticles encapsulated within the CLARITY biomaterial-loaded immunoadjuvant volume injected within the tumor microenvironment. The release kinetics of the drug payload have not been particularly studied here. The sustained release of drug delivery over time is based on the MRI/CT imaging done and is due to the biodegradability of both polymers, chitosan and sodium alginate, over time. Future studies will focus on quantifying the release kinetics of both hydrophobic and hydrophilic drugs over time from the CLARITY biomaterial. The expected impacts on long-term therapeutic efficacy is that due to the bioavailability of the immunoadjuvants within the tumor microenvironment, there is a higher likelihood of eliminating cancer cells.

Manganese oxide-based nano-platforms are responsive to tumor microenvironments due to their intrinsically high hydrogen peroxide/glutathione/H^+^ concentrations, thus allowing a likely robust cytotoxic action towards cancer cells by themselves [25,30,41]. Previous work has found that there is a synergy in combining manganese oxide nanoparticles with radiotherapy that is demonstrated in alleviating tumor hypoxia thereby increasing cancer cells sensitization to radiation damage [41,42]. Manganese oxide nanoparticles actuate the transformation of H_2_O_2_ into O_2_, which can diminish tumor hypoxia and enhance the efficacy of radiotherapy [42]. In addition, there is an immune activation that is mediated by the liberation of manganese ions from the nanoparticles that can trigger the cGAS–STING pathway as a crucial immune biochemical cascade to foster the enlistment of immune cells to the tumor site, leading to an antitumor immune response [42]. A previous study has reported that manganese oxide nanoparticles are able to notably boost the efficacy of radiotherapy by modifying the tumor microenvironment through oxygenation and immune activation [42]. The CLARITY biomaterial formulated with manganese oxide nanoparticles can therefore be employed as a multifunctional fiducial that helps to boost the efficacy of the loaded drug at the tumor site by providing sustainable amounts of the drug over time within the tumor microenvironment. Future studies will evaluate any synergistic effects from the combination treatment of CLARITY biomaterial_Mn_2_O_3_-loaded immunoadjuvants and radiotherapy against any individual treatment (e.g., single treatment cohorts of immunoadjuvants or radiotherapy) by assessing possible tumor volume regression over time.

Expected advantages of CLARITY biomaterial include the following: (i) Extended Stability— Removing moisture through lyophilization can delay the degradation of the fiducial, extending its shelf life and maintaining its efficacy over time [43]; (ii) Improved Storage and Transport— Lyophilized products can often be stored and transported at higher temperatures than their non-lyophilized counterparts, reducing the need for cold chain logistics [43,44]; (iii) Decreased Volume and Weight— The lyophilization process reduces the volume and weight of the product, which can lower shipping costs and simplify handling; (iv) Commercial Viability— For certain biologics and other drugs, supply in a lyophilized or powder form requiring reconstitution at the point of delivery may be the only feasible pathway to commercial launch; (v) Reduced Contamination Risk— All lyophilization processes occur in a controlled environment, minimizing the chances of material contamination; and (vi) Storage Flexibility— Lyophilized products can be stored at higher temperatures, reducing the need for refrigeration during transportation and storage. This flexibility is critically valuable for drugs that are sensitive to temperature changes.

This study investigated for the first time this newly designed CLARITY biomaterial for its theranostic properties in regards to providing imaging contrast and enhancing the efficacy of treatment compared to the control cohort. However, future studies will focus on further characterization of the CLARITY biomaterial in terms of its physicochemical properties, stability under various storage conditions, and behavior in different biological environments. The limitations of this study consist of a lack of more detailed characterization of the CLARITY biomaterial and also varying the amount of CLARITY biomaterial used in its reconstitution with a drug to assess its imaging contrast over time. The combination of CLARITY biomaterial loaded with immunoadjuvants and radiotherapy needs to be further explored to investigate if there are any synergistic effects from this combination therapy. However, transitioning to the clinical phase of the CLARITY Biomaterial will pose several challenges, from preclinical studies to clinical applications, which include: (a) formulating CLARITY biomaterial with the pharmaceutical grade of its individual products, such as the chitosan and sodium alginate polymers or manganese oxide nanoparticles; (b) Providing extensive safety and toxicity data from small rodents to primate animals such as monkeys/macaques; and(c) optimal efficacy of the combination treatment compared to individual treatment and the control. These initial results justify future studies and in large cohorts of mice to evaluate the immunological aspects of using the immunoadjuvants. This study also serves as an impetus for future studies to investigate the effectiveness of ultra-high dose rates like FLASH RT combined with CLARITY biomaterial loaded with immunoadjuvants for different cancers. 

## 5. Conclusions

This investigation shows that CLARITY biomaterial has potential as a device that can serve as a fiducial and for drug delivery of both hydrophobic drugs like Caflanone (FBL-03G) and hydrophilic drugs such as anti-CD40 monoclonal antibody. Further research and development are underway to optimize the formulation and reconstitution process continually.

## Data Availability

The original contributions presented in the study are included in the article/Supplementary Materials, further inquiries can be directed to the corresponding authors.

## Supplementary Materials

The following supporting information can be downloaded at: https://www.mdpi.com/article/10.3390/jfb15100285/s1, Figure S1: Transmission electron images of the CLARITY Biomaterial; Figure S2: Image-guided capability of CLARITY Biomaterial.

## References

[1] Brouwer C.L., Steenbakkers R.J.H.M., Bourhis J., Budach W., Grau C., Grégoire V., van Herk M., Lee A., Maingon P., Nutting C. (2015). CT-Based Delineation of Organs at Risk in the Head and Neck Region: DAHANCA, EORTC, GORTEC, HKNPCSG, NCIC CTG, NCRI, NRG Oncology and TROG Consensus Guidelines. Radiother. Oncol..

[2] Ezzell G.A., Galvin J.M., Low D., Palta J.R., Rosen I., Sharpe M.B., Xia P., Xiao Y., Xing L., Yu C.X. (2003). Guidance Document on Delivery, Treatment Planning, and Clinical Implementation of IMRT: Report of the IMRT Subcommittee of the AAPM Radiation Therapy Committee. Med. Phys..

[3] Kestin L.L., Sharpe M.B., Frazier R.C., Vicini F.A., Yan D., Matter R.C., Martinez A.A., Wong J.W. (2000). Intensity Modulation to Improve Dose Uniformity with Tangential Breast Radiotherapy: Initial Clinical Experience. Int. J. Radiat. Oncol..

[4] Xing L., Thorndyke B., Schreibmann E., Yang Y., Li T.-F., Kim G.-Y., Luxton G., Koong A. (2006). Overview of Image-Guided Radiation Therapy. Med. Dosim..

[5] Young M.R., Yu J.B. (2016). Intensity Modulated Radiotherapy and Image Guidance. Prostate Cancer.

[6] Moningi S., Abi Jaoude J., Kouzy R., Lin D., Nguyen N.D., Garcia Garcia C.J., Phan J.L., Avila S., Smani D., Cazacu I.M. (2021). Impact of Fiducial Marker Placement Before Stereotactic Body Radiation Therapy on Clinical Outcomes in Patients With Pancreatic Cancer. Adv. Radiat. Oncol..

[7] Moskalenko M., Jones B.L., Mueller A., Lewis S., Shiao J.C., Zakem S.J., Robin T.P., Goodman K.A. (2023). Fiducial Markers Allow Accurate and Reproducible Delivery of Liver Stereotactic Body Radiation Therapy. Curr. Oncol..

[8] Saad A., Goldstein J., Lawrence Y.R., Weiss I., Saad R., Spieler B., Symon Z. (2015). Transperineal Implantation of Gold Fiducial Markers (Gold Seeds) for Prostate Image-guided Radiation Therapy: A Feasible Technique Associated with a Low Risk of Complications. J. Med. Radiat. Sci..

[9] de Blanck S.R., Scherman-Rydhög J., Siemsen M., Christensen M., Baeksgaard L., Irming Jølck R., Specht L., Andresen T.L., Persson G.F. (2018). Feasibility of a Novel Liquid Fiducial Marker for Use in Image Guided Radiotherapy of Oesophageal Cancer. Br. J. Radiol..

[10] Serizawa I., Kozuka T., Soyano T., Sasamura K., Kamima T., Kunogi H., Kurihara N., Numao N., Yamamoto S., Yonese J. (2024). Clinical and Dosimetric Comparison Between Non-Image Guided Radiation Therapy and Fiducial-Based Image Guided Radiation Therapy With or Without Reduced Margin in Intensity Modulated Radiation Therapy for Prostate Cancer. Adv. Radiat. Oncol..

[11] Camus M., Karsenti D., Levy J., Moreno M., Coron E., Esch A., Williet N., Wangermez M., Koch S., Valats J.C. (2024). Success Rate of Fiducial Markers Placement for Treatment of Esophageal or Rectal Cancers, a Prospective Multicenter Study (FIDECHO Study). Gastrointest. Endosc..

[12] Sarma G., Kashya H., Medhi P.P., Kalita R., Lahkar D. (2024). Unravelling the Landscape of Image-Guided Radiotherapy: A Comprehensive Overview. Palliat. Med. Pract..

[13] Sengupta C., Nguyen D.T., Moodie T., Mason D., Luo J., Causer T., Liu S.F., Brown E., Inskip L., Hazem M. (2024). The First Clinical Implementation of Real-Time 6 Degree-of-Freedom Image-Guided Radiotherapy for Liver SABR Patients. Radiother. Oncol..

[14] Azzarouali S., Goudschaal K., Visser J., Bel A., Daniëls L., den Boer D. (2024). Cone-Beam Computed Tomography-Guided Online Adaptive Radiotherapy: Promising Results for Bladder Cancer Case. Cureus.

[15] Moreau M., Acter S., Ngema L.M., Bih N., Sy G., Keno L.S., Chow K.F., Sajo E., Nebangwa O., Walker J. (2023). Pre-Clinical Investigations of the Pharmacodynamics of Immunogenic Smart Radiotherapy Biomaterials (ISRB). Pharmaceutics.

[16] Opbroek T.J.S., Willems Y.C.P., Verhaegen F., de Ridder R., Hoge C., Melenhorst J., Bakers F., Grabsch H.I., Buijsen J., Van Limbergen E.J. (2023). BioXmark^®^ Liquid Fiducials to Enable Radiotherapy Tumor Boosting in Rectal Cancer, a Feasibility Trial. Clin. Transl. Radiat. Oncol..

[17] Moreau M., Richards G., Yasmin-Karim S., Narang A., Deville C., Ngwa W. (2022). A Liquid Immunogenic Fiducial Eluter for Image-Guided Radiotherapy. Front. Oncol..

[18] Moreau M., Keno L.S., China D., Mao S., Acter S., Sy G., Hooshangnejad H., Chow K.F., Sajo E., Walker J. (2024). Investigating the Use of a Liquid Immunogenic Fiducial Eluter Biomaterial in Cervical Cancer Treatment. Cancers.

[19] Ngwa W., Boateng F., Kumar R., Irvine D.J., Formenti S., Ngoma T., Herskind C., Veldwijk M.R., Hildenbrand G.L., Hausmann M. (2017). Smart Radiation Therapy Biomaterials. Int. J. Radiat. Oncol..

[20] Soni K.S., Desale S.S., Bronich T.K. (2016). Nanogels: An Overview of Properties, Biomedical Applications and Obstacles to Clinical Translation. J. Control Release.

[21] Tripathy S., Verma D.K., Gupta A.K., Srivastav P.P., Patel A.R., Chávez González M.L., Utama G.L., Aguilar C.N. (2023). Nanoencapsulation of Biofunctional Components as a Burgeoning Nanotechnology-Based Approach for Functional Food Development: A Review. Biocatal. Agric. Biotechnol..

[22] Xu F., Zhu J., Lin L., Zhang C., Sun W., Fan Y., Yin F., van Hest J.C.M., Wang H., Du L. (2020). Multifunctional PVCL Nanogels with Redox-Responsiveness Enable Enhanced MR Imaging and Ultrasound-Promoted Tumor Chemotherapy. Theranostics.

[23] Cho M., Sethi R., Ananta narayanan J.S., Lee S.S., Benoit D.N., Taheri N., Decuzzi P., Colvin V.L. (2014). Gadolinium Oxide Nanoplates with High Longitudinal Relaxivity for Magnetic Resonance Imaging. Nanoscale.

[24] Sun W., Thies S., Zhang J., Peng C., Tang G., Shen M., Pich A., Shi X. (2017). Gadolinium-Loaded Poly(N -Vinylcaprolactam) Nanogels: Synthesis, Characterization, and Application for Enhanced Tumor MR Imaging. ACS Appl. Mater. Interfaces.

[25] Yang G., Xu L., Chao Y., Xu J., Sun X., Wu Y., Peng R., Liu Z. (2017). Hollow MnO2 as a Tumor-Microenvironment-Responsive Biodegradable Nano-Platform for Combination Therapy Favoring Antitumor Immune Responses. Nat. Commun..

[26] Hsu B.Y.W., Kirby G., Tan A., Seifalian A.M., Li X., Wang J. (2016). Relaxivity and Toxicological Properties of Manganese Oxide Nanoparticles for MRI Applications. RSC Adv..

[27] Martinez de la Torre C., Grossman J.H., Bobko A.A., Bennewitz M.F. (2020). Tuning the Size and Composition of Manganese Oxide Nanoparticles through Varying Temperature Ramp and Aging Time. PLoS ONE.

[28] Nie D., Zhu Y., Guo T., Yue M., Lin M. (2022). Research Advance in Manganese Nanoparticles in Cancer Diagnosis and Therapy. Front. Mater..

[29] Liu Z., Zhang S., Lin H., Zhao M., Yao H., Zhang L., Peng W., Chen Y. (2018). Theranostic 2D Ultrathin MnO_2_ Nanosheets with Fast Responsibility to Endogenous Tumor Microenvironment and Exogenous NIR Irradiation. Biomaterials.

[30] Bonet-Aleta J., Calzada-Funes J., Hueso J.L. (2022). Manganese Oxide Nano-Platforms in Cancer Therapy: Recent Advances on the Development of Synergistic Strategies Targeting the Tumor Microenvironment. Appl. Mater. Today.

[31] Ding B., Zheng P., Ma P., Lin J. (2020). Manganese Oxide Nanomaterials: Synthesis, Properties, and Theranostic Applications. Adv. Mater..

[32] Siegel R.L., Giaquinto A.N., Jemal A. (2024). Cancer Statistics, 2024. CA Cancer J. Clin..

[33] Dizon D.S., Kamal A.H. (2024). Cancer Statistics 2024: All Hands on Deck. CA Cancer J. Clin..

[34] Kenner B.J., Chari S.T., Maitra A., Srivastava S., Cleeter D.F., Go V.L.W., Rothschild L.J., Goldberg A.E. (2016). Early Detection of Pancreatic Cancer—A Defined Future Using Lessons From Other Cancers. Pancreas.

[35] Bekkali N.H., Oppong K. (2017). Pancreatic Ductal Adenocarcinoma Epidemiology and Risk Assessment: Could We Prevent? Possibility for an Early Diagnosis. Endosc. Ultrasound.

[36] Yu J., Blackford A.L., dal Molin M., Wolfgang C.L., Goggins M. (2015). Time to Progression of Pancreatic Ductal Adenocarcinoma from Low-to-High Tumour Stages. Gut.

[37] He R., Jiang W., Wang C., Li X., Zhou W. (2024). Global Burden of Pancreatic Cancer Attributable to Metabolic Risks from 1990 to 2019, with Projections of Mortality to 2030. BMC Public Health.

[38] Silverman D.T., Hoover R.N., Brown L.M., Swanson G.M., Schiffman M., Greenberg R.S., Hayes R.B., Lillemoe K.D., Schoenberg J.B., Schwartz A.G. (2003). Why Do Black Americans Have a Higher Risk of Pancreatic Cancer than White Americans?. Epidemiology.

[39] Hong S.S., Bae S.H., Hwang J., Lee E.J. (2024). Transperineal versus Transrectal Prostate Fiducial Insertion in Radiation Treatment of Prostate Cancer: A Systematic Review and Meta-Analysis. Ultrasonography.

[40] Cendales R., Torres F., Arbelaez J., Gaitan A., Vasquez J., Bobadilla I. (2015). Displacements of Fiducial Markers in Patients with Prostate Cancer Treated with Image Guided Radiotherapy: A Single-Institution Descriptive Study. Rep. Pract. Oncol. Radiother..

[41] Liu X., Kifle M.T., Xie H., Xu L., Luo M., Li Y., Huang Z., Gong Y., Wu Y., Xie C. (2022). Biomineralized Manganese Oxide Nanoparticles Synergistically Relieve Tumor Hypoxia and Activate Immune Response with Radiotherapy in Non-Small Cell Lung Cancer. Nanomaterials.

[42] Huang Y., Ruan Y., Ma Y., Chen D., Zhang T., Fan S., Lin W., Huang Y., Lu H., Xu J.-F. (2023). Immunomodulatory Activity of Manganese Dioxide Nanoparticles: Promising for Novel Vaccines and Immunotherapeutics. Front. Immunol..

[43] Damiri F., Bachra Y., Bounacir C., Laaraibi A., Berrada M. (2020). Synthesis and Characterization of Lyophilized Chitosan-Based Hydrogels Cross-Linked with Benzaldehyde for Controlled Drug Release. J. Chem..

[44] Suhail M., Fang C.-W., Chiu I.-H., Khan A., Wu Y.-C., Lin I.-L., Tsai M.-J., Wu P.-C. (2023). Synthesis and Evaluation of Alginate-Based Nanogels as Sustained Drug Carriers for Caffeine. ACS Omega.

