# Supplementary material for: Customizable Lyophilized Agent for Radiotherapy Imaging and TherapY (CLARITY)
Source: J Funct Biomater. 2024 Sep 27;15(10):285. doi: 10.3390/jfb15100285 (PMC11508613; doi:10.3390/jfb15100285)
Supplement: Supplementary file 1 [file jfb-15-00285-s001.zip › jfb-3152265-supplementary.pdf]

Journal of
Functional
Biomaterials

Article

Customizable Lyophilized Agent for Radiotherapy Imaging and TherapY (CLARITY)

Michele Moreau [1,*], Debarghya China [1,2], Gnagna Sy [1,2], Kai Ding [1] and Wilfred Ngwa [1,*]

[1] Department of Radiation Oncology and Molecular Radiation Sciences, Johns Hopkins University School of Medicine, Baltimore, MD 21287, USA; dchina1@jhmi.edu (D.C.); gsy1@jhu.edu (G.S.); kai@jhu.edu (K.D.)

[2] Department of Biomedical Engineering, Johns Hopkins University School of Medicine, Baltimore, MD 21218, USA

* Correspondence: mmoreau1@jh.edu (M.M.); wngwa1@jhmi.edu (W.N.)

S1. Transmission Electron Images of the CLARITY Biomaterial

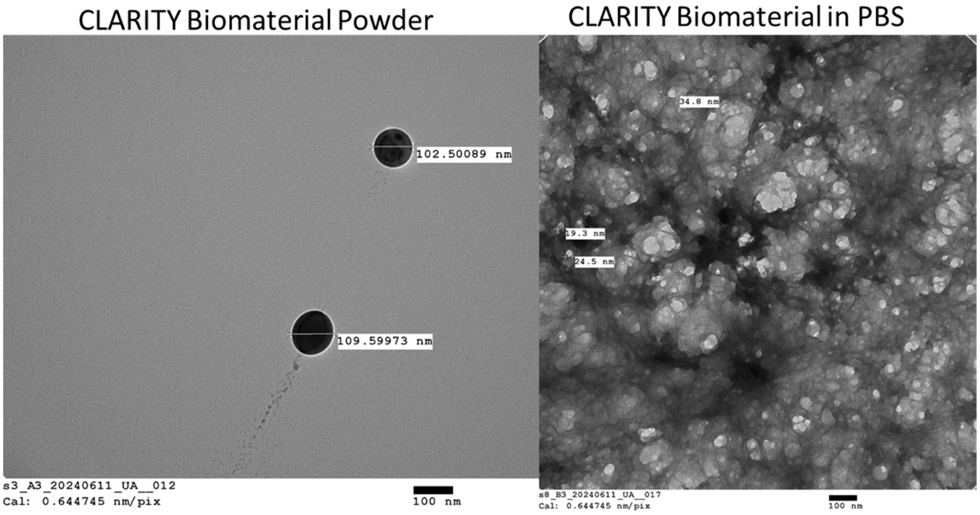

(a)

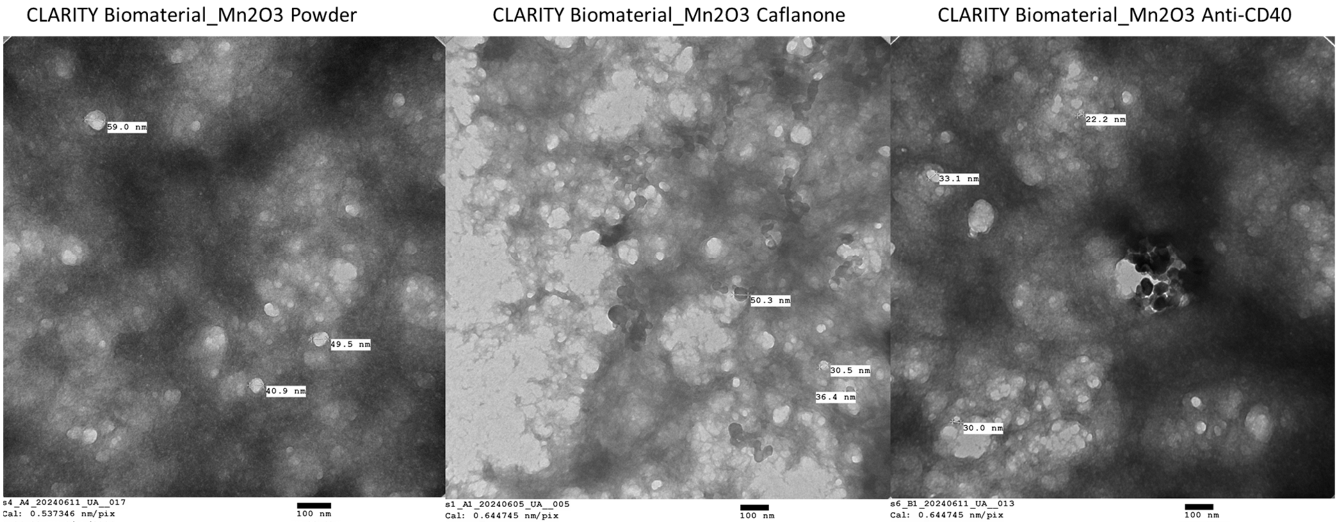

(b)

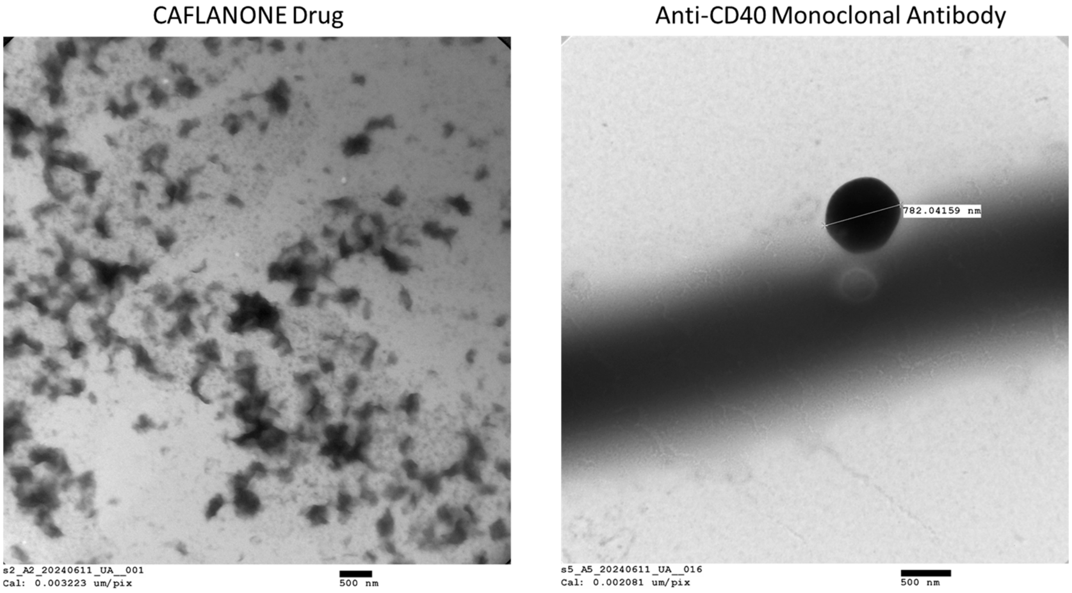

(c)

Figure S1. Transmission electron microscopic images CLARITY Biomaterial. a) The morphology and size of CLARITY Biomaterial formulated without any nanoparticles in powder and suspension forms are presented (Scale = 100nm). b) CLARITY Biomaterial formulated with manganese oxide nanoparticles unloaded and loaded with either caflanone or anti-CD40 drug are exhibited highlighting similar sizes (Scale = 100nm). c) The morphology of the free drugs either caflanone or anti-CD40 is shown (scale = 500nm).

S2. Image-guided capability of CLARITY Biomaterial

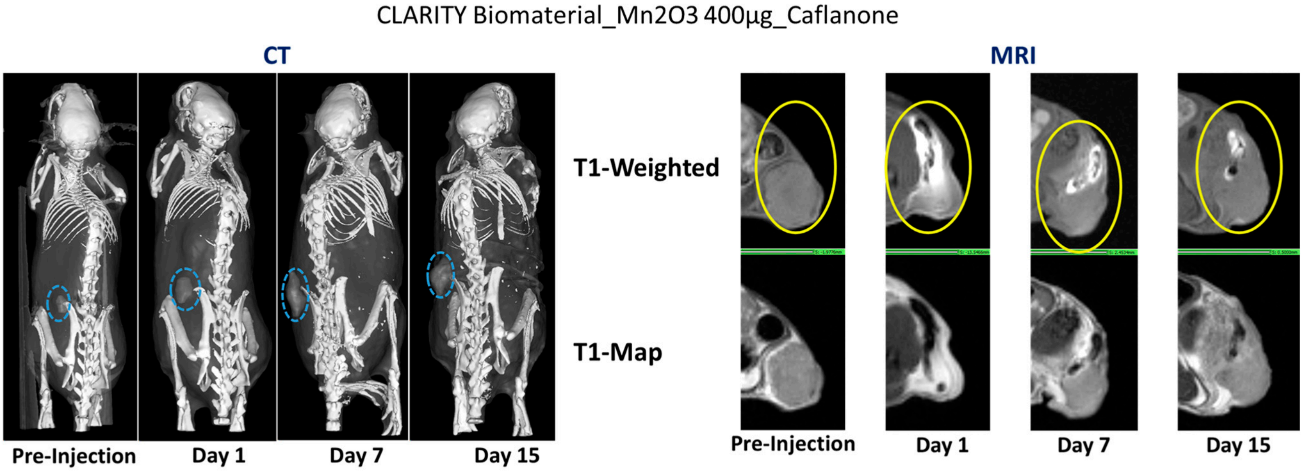

(a)

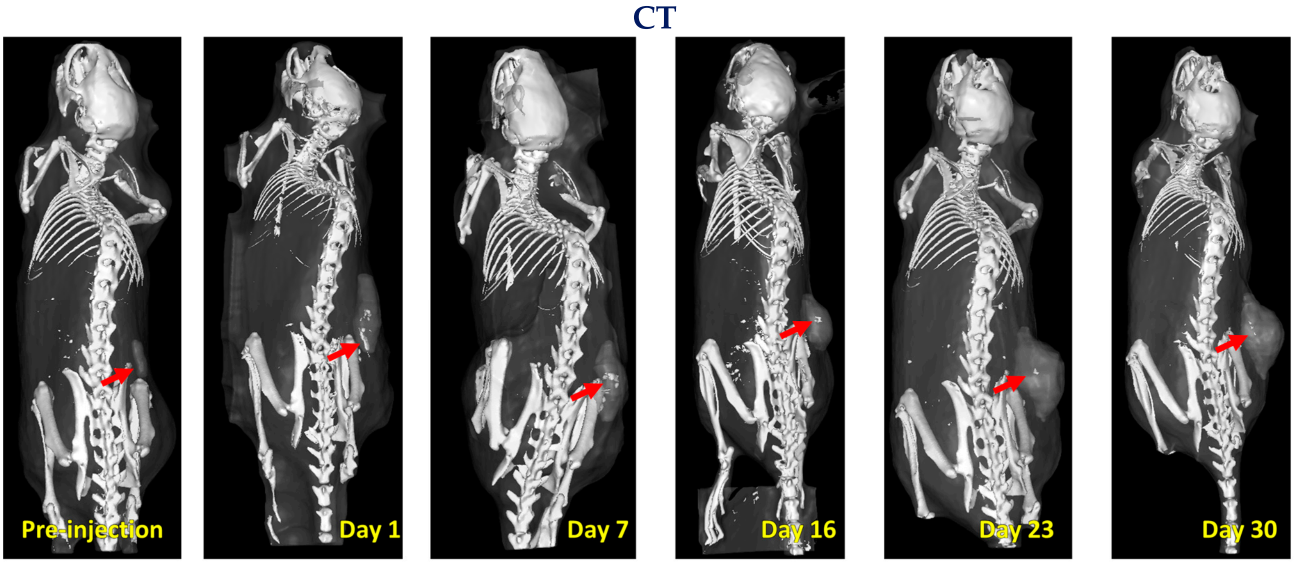

(b)

Figure S2. IGRT of CLARITY Biomaterial formulated with Mn2O3. Mass of the CLARITY Biomaterial_Mn2O3 used to reconstitute with Caflanone was varied where for a) m = 5mg and b) m = 20mg. a) Pancreatic tumored mouse injected intratumorally with CLARITY Biomaterial_Mn2O3 loaded with 400μg of Caflanone drug. CT contrast is not seen within the tumor region highlighted in the dotted blue circle whereas MRI contrast is observed up to 15 days post treatment as indicated by the white signal within the tumor region encircled in the solid yellow circle in the T1-weghted images. b) CT images are displayed for up to 30-days post treatment where contrast as indicated b the red arrow has been observed within the tumor region for up to 30-days post treatment. The CLARITY Biomaterial_Mn2O3 is loaded with 800μg of Caflanone drug.
